# Prognostic utility of rhythmic components in 24-h ambulatory blood pressure monitoring for the risk stratification of chronic kidney disease patients with cardiovascular co-morbidity

**DOI:** 10.1038/s41371-023-00884-0

**Published:** 2024-01-11

**Authors:** Nadim El Jamal, Thomas G. Brooks, Jordana Cohen, Raymond R. Townsend, Giselle Rodriguez de Sosa, Vallabh Shah, Chronic Renal Insufficiency Cohort Study (CRIC) Consortium, Robert G. Nelson, Paul E. Drawz, Panduranga Rao, Zeenat Bhat, Alexander Chang, Wei Yang, Garret A. FitzGerald, Carsten Skarke

**Affiliations:** 1 Institute for Translational Medicine and Therapeutics (ITMAT), University of Pennsylvania Perelman School of Medicine, Philadelphia, PA USA; 2 Renal-Electrolyte and Hypertension Division, University of Pennsylvania Perelman School of Medicine, Philadelphia, PA USA; 3 Department of Biostatistics, Epidemiology and Informatics, University of Pennsylvania Perelman School of Medicine, Philadelphia, PA USA; 4 Department of Internal Medicine, School of Medicine, University of New Mexico, Albuquerque, NM USA; 5 Department of Biochemistry, School of Medicine, University of New Mexico, Albuquerque, NM USA; 6 The Chronic Kidney Disease Section, National Institute of Diabetes and Digestive and Kidney Diseases, National Institutes of Health, Phoenix, AZ USA; 7 Division of Nephrology and Hypertension, University of Minnesota, Minneapolis, MN USA; 8 Department of Internal Medicine, University of Michigan, Ann Arbor, MI USA; 9 Kidney Health Research Institute, Department of Population Health Sciences, Geisinger, Danville, PA USA; 10 Department of Medicine, University of Pennsylvania Perelman School of Medicine, Philadelphia, PA USA

**Keywords:** Risk factors, Hypertension, Chronic kidney disease

## Abstract

Chronic kidney disease (CKD) represents a significant global burden. Hypertension is a modifiable risk factor for rapid progression of CKD. We extend the risk stratification by introducing the non-parametric determination of rhythmic components in 24-h profiles of ambulatory blood pressure monitoring (ABPM) in the Chronic Renal Insufficiency Cohort (CRIC) and the African American Study for Kidney Disease and Hypertension (AASK) cohort using Cox proportional hazards models. We find that rhythmic profiling of BP through JTK_CYCLE analysis identifies subgroups of CRIC participants that were more likely to die due to cardiovascular causes. While our fully adjusted model shows a trend towards a significant association between absent cyclic components and cardiovascular death in the full CRIC cohort (HR: 1.71,95% CI: 0.99–2.97, *p* = 0.056), CRIC participants with a history of cardiovascular disease (CVD) and absent cyclic components in their BP profile had at any time a 3.4-times higher risk of cardiovascular death than CVD patients with cyclic components present in their BP profile (HR: 3.37, 95% CI: 1.45–7.87, *p* = 0.005). This increased risk was not explained by the dipping or non-dipping pattern in ABPM. Due to the large differences in patient characteristics, the results do not replicate in the AASK cohort. This study suggests rhythmic blood pressure components as a potential novel biomarker to unmask excess risk among CKD patients with prior cardiovascular disease.

## Introduction

Chronic kidney disease (CKD) is a significant global burden, with a prevalence of 9011 cases per 100,000 people. For 2019, this translated into more than 1.4 million deaths globally [1]. Contributing to that burden is the increasing incidence of major cardiovascular events as kidney function worsens [2]. Well-controlled blood pressure can mitigate these risks, and the International Society of Nephrology blood pressure guidelines suggest the use of ambulatory blood pressure monitoring (ABPM) to complement in-office blood pressure measurements in CKD patients (level 2B recommendation) [3].

A particular advantage of ABPM is the ability to capture blood pressure variability over the course of 24 h (diurnal rhythms) in a patient’s home environment. Oscillatory signals are abundantly detected in physiologic parameters collected from healthy humans under normal living conditions [4]. Oscillations are evident in parameters of renal function, including glomerular filtration rate (GFR), renal blood flow, and electrolyte excretion [5]. Whether deconsolidation of diurnal rhythms contributes to disease expression is of interest in CKD. Loss of renal function leads, for example, to increased sleep fragmentation and conversion of the nocturnal dip in the diurnal blood pressure profile into a non-dipping type which is associated with higher mortality (reviewed by Mohandas et al) [6]. In our analysis of the UK Biobank, lower daily rhythms of wrist temperature were associated with a higher prevalence of renal failure [7].

There are many methods to study variability in ABPM recordings. Among these are dispersion methods, methods assessing beat-to-beat variability and methods quantifying time-specific variations [8]. Diurnal variability in blood pressure is commonly approached as a binary variable, i.e. presence or absence of the nocturnal dip. The absence of the nocturnal dip, and more so the “inverse dipper” with a rise in blood pressure at night, may be associated with worse renal outcomes in CKD patients [9]. However, a limitation is that definitions of the nocturnal dip are variable across studies and explore little the characteristics of a time series data set, though comprehensive time-specific assessments are available from adjacent fields. One such method is the JTK_CYCLE algorithm, a non-parametric test to detect the presence of diurnal rhythms primarily in time-integrated gene expression studies [10]. Here, we adopted the JTK_CYCLE algorithm to parse blood pressure rhythms beyond those detected by the dipping phenotype, and to assess the prognostic utility in ABPM for risk stratification of CKD patients. JTK_CYCLE was designed as a computationally efficient algorithm that detects parameters that cycle across the 24-h day. This algorithm measures how closely a time series of measurements rises steadily to a peak and descends steadily to a trough. Precedence for the JTK_CYCLE based analysis of time series blood pressure measurements comes from the preclinical domain. Blood pressure rhythmic components were absent in mice lacking the core clock gene BMAL1 (brain and muscle ARNT-like protein 1) in their smooth muscles compared to littermate controls, suggesting that peripheral molecular clocks contribute to blood pressure regulation [11]. We hypothesized that JTK_CYCLE extracts distinct features in blood pressure time series which are neither picked up by the dipping ratio (which measures the magnitude of decrease in the mean nighttime blood pressure to the mean day time blood pressure) nor by measures of “beat-to-beat” variability such as average-real variability (ARV, which quantifies the dispersion across successive observations) and thus could complement dipping ratio and ARV as an additional index for risk stratifying patients.

## Methods

### Cohorts

We obtained data from two prospective observational cohort studies of CKD patients; the Chronic Renal Insufficiency Cohort (CRIC) and the African American Study for Hypertension and Kidney Disease (AASK) cohort study. The data were made available upon request from the CRIC publication executive committee and from the NIDDK-CR (a program from the National Institute of Diabetes and Digestive and Kidney Diseases), respectively.

The CRIC study is an ongoing multicenter observational study that enrolled adult patients with various etiologies of chronic kidney disease. Detailed methods have been previously published [12]. As an ancillary study, participants were randomly chosen from the different clinical sites to undergo ABPM, of whom 1502 provided valid ABPM recordings [9]. We based our analysis of the CRIC cohort on these participants. Participants who were night-shift workers and who had previous end-stage renal disease (ESRD) were excluded and ABPM was set to take measurements every 30 min during the day and night. Further details on participant selection and ABPM measurement were previously published [9]. Outcomes of interest in the CRIC study are a composite cardiovascular outcome of congestive heart failure incidence or events, myocardial infarction, stroke, or peripheral artery disease, cardiovascular death, all-cause death, and a composite renal outcome of ESRD or a 50% decline in eGFR. Outcomes were ascertained by asking participants about hospitalizations. Hospital records were then obtained and adjudicated by two clinicians using predefined guidelines [9].

The rationale and design of the AASK cohort study have been previously published. In brief, The AASK cohort study is a prospective multicenter observational study that is an extension of the AASK trial which tested the effects of a low mean arterial pressure (MAP) goal and the use of antihypertensives on renal function in African Americans with Hypertensive Kidney Disease. In the AASK Cohort study, trial participants who did not reach end-stage renal disease were followed up for five years after trial conclusion with the main outcome of interest being a composite renal outcome defined as a doubling of serum creatinine from baseline, ESRD or Death. A cardiovascular outcome was predefined as a combination of fatal or nonfatal cardiovascular events of myocardial infarction, hospitalization for heart failure, or strokes. Outcomes were ascertained from participants at each contact. Hospitalization records were reviewed by a cardiovascular event committee to adjudicate cardiovascular outcomes. Participants had their blood pressure managed by the AASK investigators with a systolic blood pressure goal of less than 130 mmHg and a diastolic blood pressure goal of less than 80 mmHg. Investigators were encouraged to follow a standardized algorithm in their pharmacologic management. 24-h ABPM with measurements every 30 min during both day and night were collected at the post-trial baseline and then every other year to total three ABPM measurements over the span of the five years of the study.

### Statistical Analysis

For both studies we considered ABPM recordings with at least 14 readings between 6 am and midnight and at least six readings between midnight and 6 am to be valid. ABPM systolic blood pressure values from CRIC and AASK cohorts were binned, for each participant, by the hour of the day and the mean values for each hour were taken. When subjects had more than 24 h of recording, corresponding hours of all days were binned together. Using Nitecap (nitecap.org) [13], a web-based tool for the rhythmic analysis of biologic parameters, we ran a JTK_CYCLE analysis on these hourly means. The JTK_CYCLE algorithm is a non-parametric test based on the Kendall tau statistic and operates by performing multiple Jonckheere–Terpstra tests. The Jonckheere–Terpstra test is a non-parametric method similar to the Kruskal–Wallis test but with an assumption of a known ordering of the medians of each group. JTK uses this to compare the actual data’s ordering to the ordering that would have been observed if the data exactly followed a cosine curve. This is then repeated for a library of cosine curves of varying phases followed by a correction for the multiple testing. This results in a P-value that indicates the presence or absence of rhythmic components in the blood pressure curve [10]. Using this P-value we categorized participants as possessing rhythmic components in blood pressure (JTK *P*-value ≤ 0.05) or having no rhythmic components (JTK *P*-value > 0.05).

We calculated dipping ratios (DR) by dividing mean nighttime blood pressure over mean day time blood pressure. Nighttime was defined as a fixed interval between midnight and 6 am. We defined normal nocturnal dipping as a DR less than or equal to 0.9, non-dipping as a DR greater than 0.9 but less than or equal to 1, and reverse dipping as a DR greater than 1. For few analyses, non-dippers and reverse dippers were combined into one group for the interpretability and comparability of results. Assessments of blood pressure control were based on the 2017 ACC/AHA guidelines for blood pressure management [14].

To assess determinants of either dipping status or the retention of rhythmic components, we fitted multivariable logistic regression models with dipping status or retention of rhythmic components as dependent outcomes. Covariate data were collected at the ABPM visit or the visit closest to that. Because in the AASK cohort study ABPM was repeated every other year, many participants had data from three ABPM sessions. Taking advantage of the multiple measurements for the AASK cohort we ran a mixed effects logistic regression analysis to account for the correlation between measurements from the same participant.

We fitted Cox proportional hazards models to study the utility of the presence of rhythmic components in the risk stratification of chronic kidney disease patients. In this analysis, baseline was determined to be the time of ABPM. Data regarding covariates used in the adjusted models were also collected at either the time of ABPM or the closest visit. For the CRIC cohort, we defined outcomes of interest to be a composite renal outcome (50% decrease in estimated glomerular filtration rate (eGFR) from baseline, or reaching end-stage renal disease), death from all causes, death from cardiovascular causes, and a composite cardiovascular outcome (myocardial infarctions, heart failure, strokes, peripheral artery disease). We also fitted Cox proportional hazards models on subgroups stratified by dipping category or the presence of prior CVD. For the AASK cohort, we defined the same outcomes as we did for the CRIC cohort except for the composite cardiovascular outcome which was defined in AASK as cardiovascular death or cardiac revascularization, nonfatal MI, heart failure hospitalization, or stroke [15]. eGFR was calculated based on the CRIC equation in the CRIC cohort and based on the CKD-EPI 2021 Creatinine equation in the AASK cohort [16, 17]. Due to the lower event rate and the smaller sample size we did not apply subgroup analyses to the AASK cohort. In survival analyses, data were censored at the end of study follow-up, withdrawal from the study and death if it is not the outcome of interest. Statistical analyses were performed using STATA 17, IBM SPSS version 26, and R 4.2.

## Results

### Cohort characteristics at the time of ABPM

ABPM recordings that passed quality control criteria were available from *n* = 1502 CRIC and *n* = 643 AASK participants. In the AASK cohort, 55.2% (355/643) of the participants completed three valid ABPM sessions that were on average two years apart. A total of 57 CRIC recordings and 16 AASK recordings which did not pass quality control criteria were excluded. The population structure was different for the two cohorts. The CRIC cohort enrolled diverse kidney disease etiologies and aimed for participants with diabetes to account for half of their cohort, while the AASK trial focused only on hypertensive kidney disease patients and excluded those with diabetes in the earlier trial phase. This is reflected by 8.6 mmHg and 10.3 mmHg higher systolic and diastolic blood pressure levels, respectively, in AASK compared to CRIC, and a lower prevalence of diabetes in former. The AASK cohort also had a higher prevalence of CVD, and a mean eGFR which was 10 mL/min lower compared to CRIC (Table 1). Rhythmic components were present in 34% (514/1502) of CRIC and 26% (169/643) of AASK participants (Table S1). Among CRIC and AASK participants categorized as non-dippers, rhythmic components were still detectable in approximately one in seven participants amounting to 16.1% (92/572) and 14.3% (38/266), respectively.

**Table 1:** Characteristics of participants in the two cohorts.

	CRIC (*n* = 1502)	AASK (*n* = 643)
Clinic systolic pressure (mmHg)	126 ± 20	133 ± 20
Clinic diastolic pressure (mmHg)	69 ± 12	79 ± 11
Having prior CVD	585 (39%)	286 (63%)
Diabetic	744 (50%)	57 (13%)
eGFR (mL/min/1.73m2)	46.02 ± 20.31	36.06 ± 14.30
Urine protein/creatinine ratio (mg/g), median (25%–75%)	155 (68–604)	64 (29–302)
Sex (Female)	661 (44%)	244(38%)
Age (years)	63.07 ± 10.27	60.47 ± 10.13
BMI (Kg/m^2^)	31.50 ± 6.94	31.14 ± 7.01
Race (Black)	582 (39%)	643 (100%)

The summary statistics are based on data collected at the time of ABPM or the closest visit.

Data presented as mean ± S.D. or number (%) unless stated otherwise.

### JTK_CYCLE, Dipping Ratio and ARV describe different components of the ABPM Curve

**Fig. 1:**
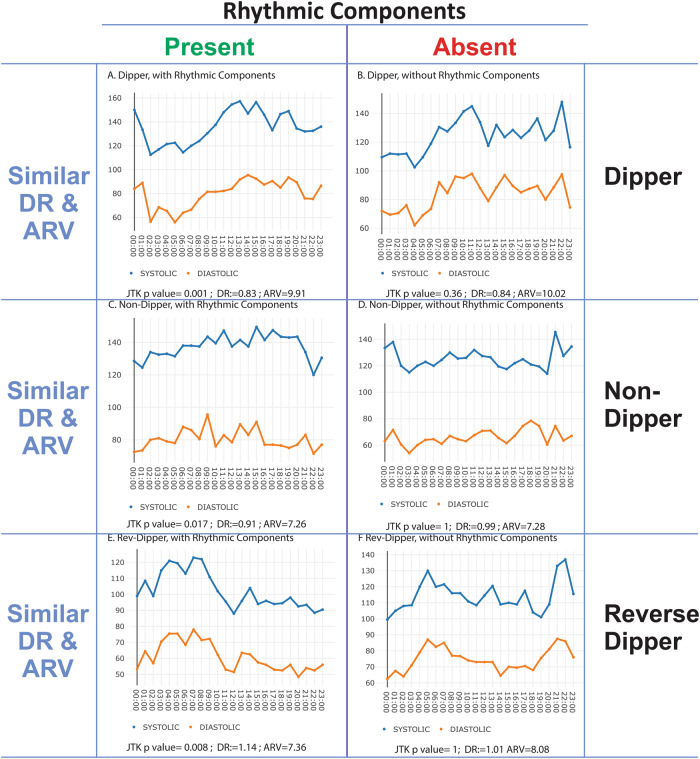
Parsing rhythmic components in 24 h blood pressure profiles. Juxtaposition of 24 h blood pressure curves and blood pressure variability metrics from six CRIC participants to illustrate the discriminatory characteristics on the basis of the JTK_CYCLE analysis while DR (Dipping Ratio) and ARV (Average real variability) show similar values between pairs of dippers in (**A**) and (**B**), non-dippers in (**C**) and (**D**), and reverse dippers in (**E**) and (**F**).

Blood pressure curves from several CRIC participants illustrate how time series blood pressure measurements with similar DR and ARV indices are discriminated by rhythmic components quantified by the JTK_CYCLE algorithm (Fig. 1). Two patients with a dipping blood pressure phenotype, DR = 0.83 and 0.84 in Fig. 1A, B, respectively, have a similar ARV of 9.91 and 10.02, respectively, but diverge on the JTK *p-value* of 0.001 compared to 0.36, respectively. Similar juxtaposition is presented for a pair of participants with a non-dipping BP phenotype in Fig. 1C, D, and for reverse dipping in Fig. 1E compared to Fig. 1F. This exemplifies that rhythmic profiling of the 24-h blood pressure curves can discriminate patients with similar dipping ratios and average-real variability indices. Next, we were interested to determine if this novel approach to parse blood pressure variability has prognostic value.

### Cardiovascular and renal risk factors associated with absence of rhythmic components

Diabetes (*p* = 0.007), African American race (*p* = 0.03), or a history of cardiovascular disease (*p* = 0.001) were associated with absent ABPM rhythmic components in CRIC (Table 2). These covariates did not reach significance in the mixed effects logistic regression applied to the AASK cohort, but BMI greater than 30 (*p* = 0.033) was significantly associated here with the absence of rhythmic components in 24-h blood pressure profiles (Table 3). The association of dipping category and the presence of rhythmic components with different risk factors further indicates these measures capture different populations and set forth the expectation that they might have different associations with health outcomes.

**Table 2:** Multivariate logistic regression for the absence of rhythmic components in blood pressure among CRIC cohort participants.

Covariate	Odds ratio	95% CI lower	95% CI upper	*P*-value
Age < 45	Reference			
Age from 45 to <65	1.27	0.78	2.07	0.329
Age 65+	1.47	0.9	2.41	0.123
BMI < 25	Reference			
BMI 25 to <30	0.87	0.61	1.26	0.465
BMI ≥ 30	1.24	0.71	1.76	0.233
eGFR > 60 ml/min/1.73 m^2^	Reference			
eGFR: 30 to <60 ml/min/1.73 m^2^	1.05	0.77	1.42	0.769
eGFR < 30 ml/min/1.73 m^2^	1.01	0.74	1.63	0.65
Proteinuria (PCR < 150 mg/g)	Reference			
Proteinuria (PCR: 150–500 mg/g)	1.19	0.86	1.63	0.29
Proteinuria (PCR > 500 mg/g)	1.23	0.88	1.74	0.231
Controlled BP	Reference			
Uncontrolled BP (Mean 24 h BP ≥ 125/75 mmHg)	0.97	0.74	1.27	0.823
Male sex	Reference			
Female sex	1.002	0.78	1.28	0.988
Non-diabetic	Reference			
Diabetic	1.41	1.1	1.82	0.007
Race: White	Reference			
Race: Black	1.5	1.15	1.96	0.03
Race: Other	1.26	0.86	1.83	0.237
Prior CVD: No	Reference			
Prior CVD: Yes	1.58	1.22	2.05	0.001

All variables in the table were included as covariates in the model.

*eGFR* estimated glomerular filtration rate by the CRIC cohort equation, *BP* blood pressure, *PCR* urine protein to creatinine ratio.

**Table 3:** Mixed effects logistic regression for the absence of rhythmic components in blood pressure among AASK cohort participants.

Covariate	Odds ratio	95% CI lower	95% CI upper	*P*-value
Age < 45	Reference			
Age from 45 to <65	1.5	0.61	3.7	0.379
Age 65+	2.23	0.88	5.64	0.091
BMI < 25	Reference			
BMI 25 to <30	1.09	0.62	1.91	0.757
BMI ≥ 30	1.84	1.05	3.22	0.033
eGFR >60 ml/min/1.73 m^2^	Reference			
eGFR: 30 to <60 ml/min/1.73 m^2^	0.38	0.12	1.21	0.101
eGFR < 30 ml/min/1.73 m^2^	0.43	0.13	1.41	0.165
Proteinuria (PCR < 150 mg/g)	Reference			
Proteinuria (PCR: 150–500 mg/g)	1.43	0.85	2.4	0.176
Proteinuria (PCR > 500 mg/g)	1.65	0.98	2.78	0.062
Controlled BP	Reference			
Uncontrolled BP (Mean 24 h BP ≥ 125/75 mmHg)	1.04	0.66	1.65	0.862
Male sex	Reference			
Female sex	1.18	0.77	1.79	0.448
Non-diabetic	Reference			
Diabetic	0.63	0.35	1.18	0.119
Prior CVD: No	Reference			
Prior CVD: Yes	0.95	0.52	1.43	0.792
Drug randomization group: ACE I	Reference			
Drug randomization group: Beta blocker	1.08	0.7	1.68	0.716
Drug randomization group: CCB	0.93	0.52	1.67	0.815
BP target randomization group: lower target (MAP < 92)	Reference			
BP target randomization group: usual target (MAP 102–107)	1.22	0.82	1.81	0.328
Time from initial ABPM (years)	0.91	0.82	1.01	0.076

All variables in the table were included as covariates in the model.

*eGFR* estimated glomerular filtration rate by the 2021 CKD-EPI equation, *PCR* urine protein to creatinine ratio, *BP* blood pressure, *ACE I* angiotensin converting enzyme inhibitor, *CCB* calcium channel blocker.

### Cardiovascular and renal risk factors associated with non-dipping BP

In the CRIC cohort, variables associated with nocturnal non-dipping included a protein-creatinine ratio (PCR) > 500 mg/g (*p* = 0.009), uncontrolled BP (*p* < 0.001), African American race (*p* = 0.001), and a history of cardiovascular disease (*p* < 0.001) (Table S2). A trend towards age-dependent emergence of nocturnal non-dipping was noticeable in participants older than 65 years (*p* = 0.047). Only uncontrolled BP (*p* = 0.006) and taking beta-blockers as antihypertensives during the clinical trial phase (*p* = 0.016) attained significance in the AASK cohort (Table S3). A history of diabetes (*p* = 0.048) trended to be associated.

### Absence of rhythmic BP components is associated with cardiovascular death in CKD patients with prior cardiovascular disease

**Fig. 2:**
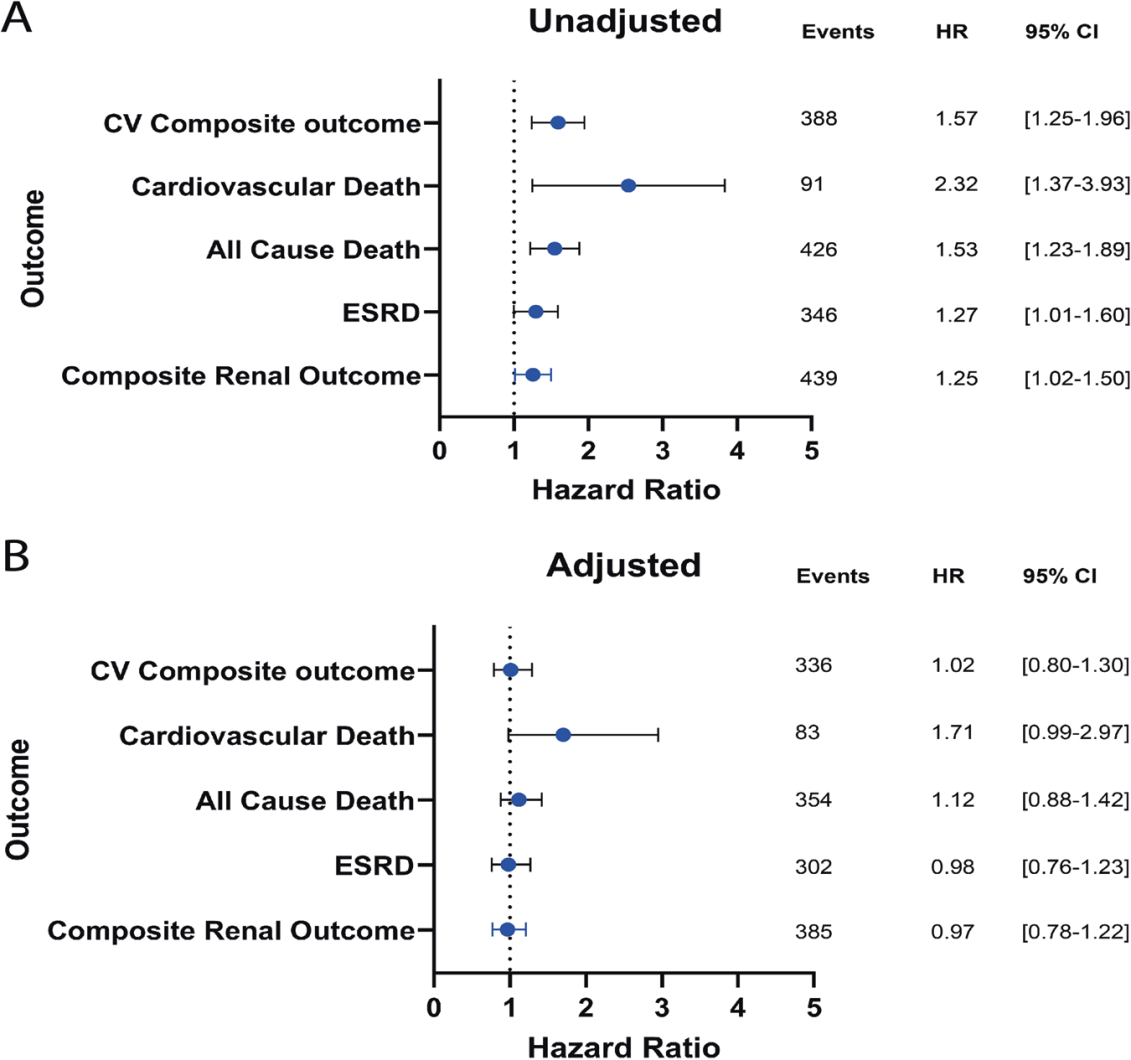
Cox proportional hazards models in the CRIC cohort. Hazard ratios for the absence of rhythmic components (JTK *p*-value > 0.05) and reaching different outcomes in the CRIC cohort as compared to the retention of rhythmic components (JTK *p*-value ≤ 0.05). **A** Unadjusted model. **B** Adjusted for Age, BMI, Sex, Diabetes, Race, eGFR, Urine Protein to Creatinine Ratio, Clinic SBP, Prior CVD.

**Fig. 3:**
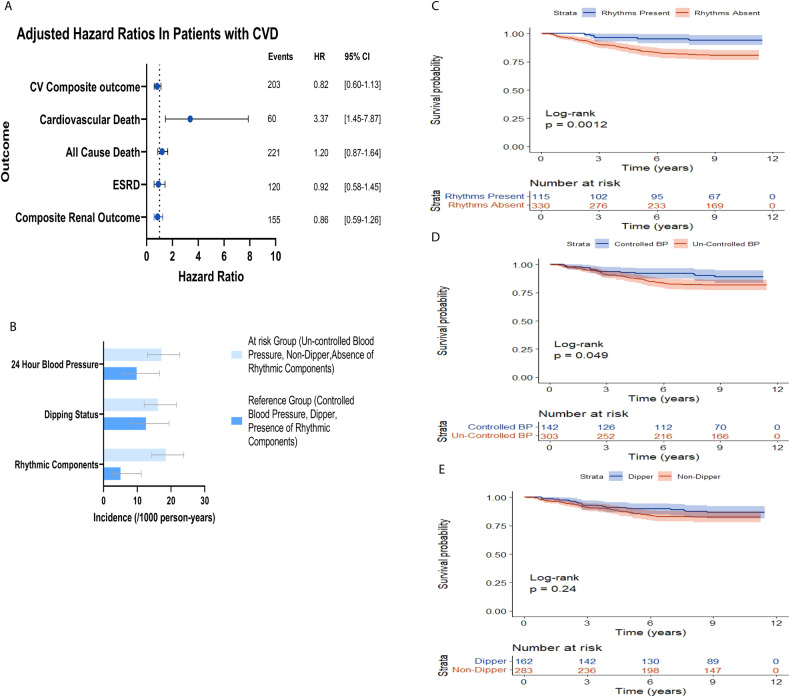
The association between cardiovascular death and the absence of rhythmic components in CRIC patients with established CVD. **A** Hazard ratios for the absence of rhythmic components (JTK *p*-value > 0.05) and reaching different outcomes in the CRIC cohort as compared to the retention of rhythmic components (JTK *p*-value ≤ 0.05) in participant with prior CVD. Adjusted for Age, BMI, Sex, Diabetes, Race, eGFR, Urine Protein to Creatinine Ratio, Clinic SBP. **B** Incidence of cardiovascular mortality in patients with prior cardiovascular disease stratified by the different ambulatory blood pressure monitoring derived parameters. Kaplan Meier survival estimates for cardiovascular death in CRIC patients with prior CVD stratified by categorizations of the different ambulatory blood pressure parameters. **C** stratified by the presence of rhythmic components. **D** stratified by blood pressure control (24 h blood pressure mean cutoff of 125/75). **E** Stratified by dipping status (Dipping ratio cutoff of 0.9).

Clinical outcomes were collected over a mean follow-up time of 8.4 (±2.6) years for the CRIC cohort and 4.5 (±1.1) years for the AASK cohort. In unadjusted Cox proportional hazards models, the absence of rhythmic components was significantly associated with reaching all outcomes of the CRIC cohort (Fig. 2A). This association was no longer significant when controlling for all covariates including prior cardiovascular disease (Fig. 2B). However, because 60 out of 83 patients who died due to cardiovascular causes already had prior CVD, we ran a subgroup analysis including only patients with prior CVD to test for an association beyond what is confounded by the presence of such a large risk factor for cardiovascular death. In our stratified analysis, CRIC participants with a history of CVD and absent cyclic components in their BP profile had at any time a 3.4-times higher risk to reach the outcome of cardiovascular death than participants with cyclic components present in their BP profile (HR: 3.37, 95% CI: 1.45–7.87, *p* = 0.005, Fig. 3A). Furthermore, the interaction term between the absence of rhythmic components and the presence of cardiovascular disease had an adjusted hazard ratio of 4.32 (95% CI: 1.32–14.13, *p* = 0.015)

This substantially increased risk was not driven by a specific subgroup, i.e., nocturnal dipper, non-dipper or reverse dipper (*p* > 0.1, Table 4). Rhythmic BP components were absent in all non- and reverse-dipping CRIC participants with cardiovascular death (*n* = 24 and *n* = 21, respectively). Among dippers with prior CVD succumbing to cardiovascular death, the majority (14 out of 20) had no cyclic components in their BP profiles, while 6 retained BP cycles (Table S4).

**Table 4:** Hazard ratios for the different dipping categories and reaching different outcomes among CRIC cohort participants with prior CVD Adjusted for Age, BMI, Sex, Diabetes, Race, eGFR, Urine Protein to Creatinine Ratio, Clinic SBP.

		Unadjusted model			Adjusted model		
Outcome	Dipping category	Hazard ratio	95% CI	*p*-value	Hazard ratio	95% CI	*p*-value
Composite renal outcome	Dipper	Reference					
	Non-dipper	1.63	1.14–2.34	0.008	1.16	0.77–1.74	0.482
	Reverse dipper	2.06	1.39–3.07	<0.001	1.56	1.00–2.42	0.049
ESRD	Dipper	Reference					
	Non-dipper	1.79	1.19–2.72	0.006	0.99	0.61–1.60	0.961
	Reverse dipper	2.05	1.30–3.25	0.002	1.37	0.83–2.28	0.222
All-cause death	Dipper	Reference					
	Non-dipper	1.01	0.75–1.34	0.995	0.8	0.58–1.10	0.163
	Reverse dipper	1.48	1.09–2.02	0.012	1.03	0.73–1.46	0.869
Cardiovascular death	Dipper	Reference					
	Non-dipper	1.06	0.59–1.92	0.843	1.08	0.58–2.00	0.81
	Reverse dipper	1.66	0.90–3.07	0.104	1.3	0.65–2.58	0.462
Composite CV outcome	Dipper	Reference					
	Non-dipper	0.93	0.69–1.25	0.616	0.81	0.56–1.12	0.195
	Reverse dipper	1.18	0.85–1.64	0.337	0.88	0.61–1.29	0.521

*DR* dipping ratio, *CV* cardiovascular, *CVD* cardiovascular disease, *ESRD* end-stage renal disease.

To quantify the occurrence of CV death further, we calculated the person-time rate for cardiovascular mortality among CRIC participants with prior CVD (Fig. 3B). The absence of rhythmic components had a statistically significant increase in the incidence rate compared to their presence (18.46 [14.30–23.82] per 1000 person years compared to 5.04 [2.65–11.22] per 1000 person years respectively, *p* < 0.001). This was neither seen in 24 h blood pressure control (17.20 [13.07–22.63] per 1000 person years in uncontrolled BP, 9.85 [5.83–22.63] per 1000 person years in controlled BP, *p* = 057) nor in dipping category (16.11 [12.03–21.58] per 1000 person years in non-dippers, 12.55 [8.10–19.45] per 1000 person years in dippers, *p* = 0.356). In the time-to-event analysis, survival probability was significantly lower in participants without rhythmic BP components compared to those who had them (log-rank *p* = 0.0012). Survival probability trended to be lower in participants with uncontrolled BP compared to controlled BP (log-rank *p* = 0.049), while no difference was detected for non-dippers compared to dippers (log-rank *p* = 0.24) as shown in Fig. 3C–E.

We turned to the 83 cardiovascular deaths adjudicated for the adjusted model (compared to 60 in the adjusted model restricted to CVD, Fig. 2B) to explore further whether absence of rhythmic components confers an increased risk for cardiovascular death. Strikingly, we found that reverse dippers without cyclic components trended to be at an 8-fold higher risk of dying from cardiovascular causes compared to reverse dippers with retained rhythms (HR: 7.23, 95% CI: 0.93–56.4, *p* = 0.059, Fig. S1). While the trend is large, the low number of events and the wide confidence interval slightly intersecting the line of no-effect invite caution in interpreting this result. All-cause death emerged as a significant signal (HR: 2.76, 95% CI: 1.32–5.77, *p* = 0.007). Absence of rhythmic components had little effect on cardiovascular or all-cause mortality among dippers (HR:1.3, 95% CI: 0.6–2.91, *p* = 0.456, and HR: 0.77, 95% CI: 0.54–1.11, *p* = 0.162, respectively) and non-dippers (HR:1.48, 95% CI: 0.44–4.93, *p* = 0.523, and HR: 1.25, 95% CI: 0.75–2.09, *p* = 0.395, respectively). As was previously published [9], participants with a non-dipping pattern (particularly reverse-dipping pattern) were at a higher risk for worsening kidney function (Table S5).

**Fig. 4:**
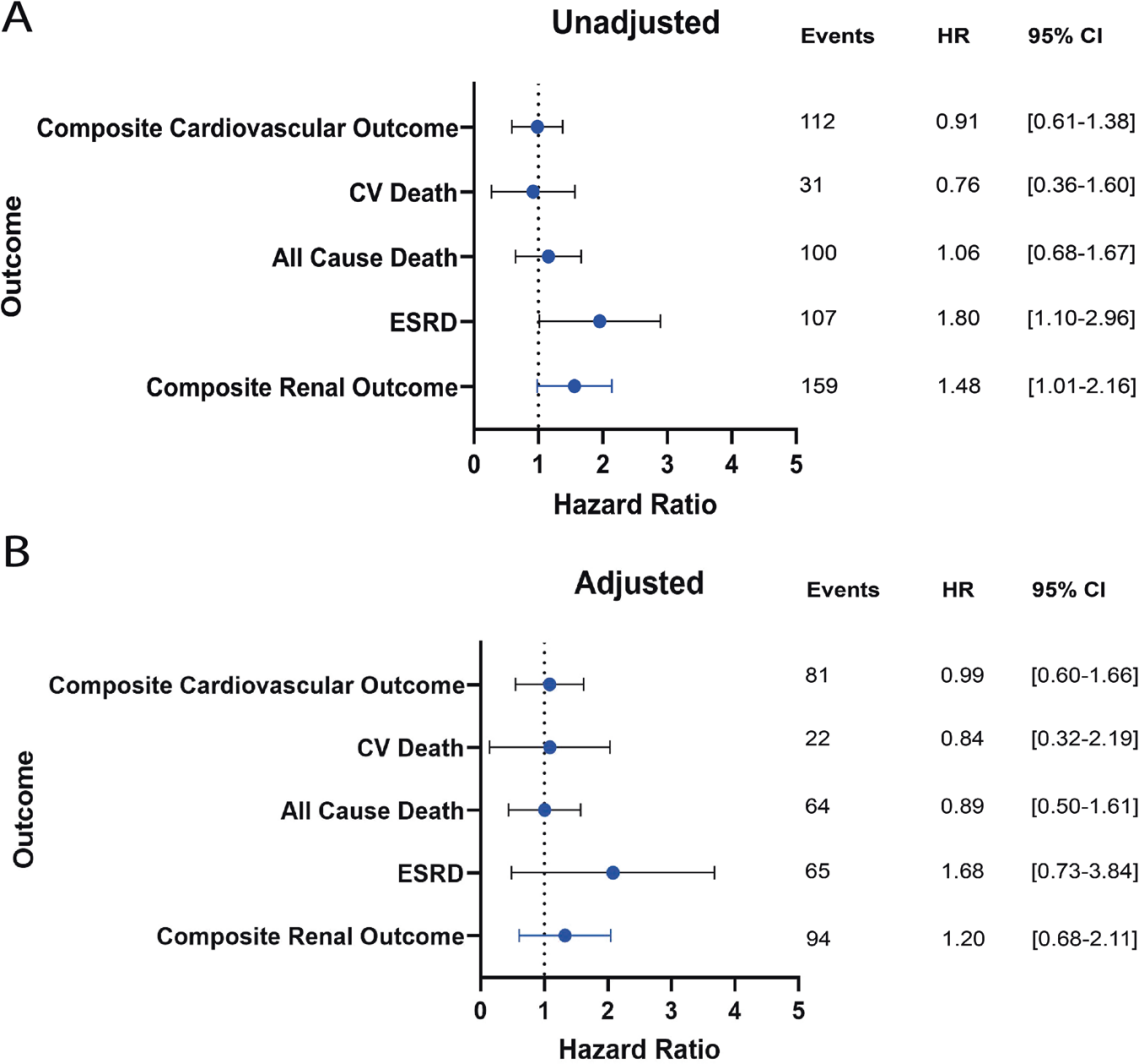
Cox proportional hazards models in the AASK cohort. Hazard ratios for the absence of rhythmic components (JTK *p*-value > 0.05) and reaching different outcomes in the AASK cohort as compared to the retention of rhythmic components (JTK *p*-value ≤ 0.05). **A** Unadjusted model. **B** Adjusted for Age, BMI, Sex, Diabetes, eGFR, Urine Protein to Creatinine Ratio, Clinic SBP, Prior CVD, Drug and blood pressure target groups randomized to in the prior trial.

For the AASK cohort, we detected in the unadjusted Cox proportional hazard model a significant association between the absence of rhythmic BP components and end-stage renal disease (HR:1.80, 95% CI: 1.10–2.96, *p* = 0.020) as well as the composite renal outcome (HR: 1.48, 95% CI: 1.01–2.16, *p* = 0.042). However, these associations lost statistical significance when fully adjusting the model (HR:1.68, 95% CI: 0.73–3.85, *p* = 0.223 and HR:1.2, 95% CI: 0.68–2.11, *p* = 0.515, respectively, Fig. 4). There were no statistically significant associations between dipping status and all outcomes in the AASK cohort except for reverse dipping and the composite cardiovascular outcome in the unadjusted model (HR: 1.79, 95% CI: 1.02–3.14, *P*-value: 0.043) (Table S6). Due to the smaller sample size and the low number of events, we did not perform subgroup analyses using the AASK cohort data.

To compare rhythmic components with non-diurnal methods of variability we fitted Cox proportional hazard models on tertials of average-real variability (ARV), a measure of sequential beat-to-beat variability. We divided the population over three tertials: ARV ≤ 9 (*n* = 579), 9 < ARV ≤ 11 (*n* = 344), or ARV > 11 (*n* = 579). We found a significant association between higher ARV tertials and all outcomes except cardiovascular death in the unadjusted model. These associations did not retain statistical significance after full adjustment (Table S7). Thus, the JTK_CYCLE algorithm is capturing a different at-risk population compared to dipping ratio or average-real-variability indices calculated from ABPM.

## Discussion

The main finding of the present study is that nonparametric characterization of time-dependent effects in ambulatory blood pressure measurements (ABPM) identified patients with chronic kidney disease at a higher likelihood of mortality irrespective of their blood pressure (BP) dipping behavior defined as nocturnal dipper, non-dipper or reverse dipper. This was evident among CRIC participants with a history of CVD where the participants without rhythmic BP components had at any time a 3.4-times higher risk to experience cardiovascular death than the participants with existing rhythmic BP components. We also found that risk factors known to increase cardiovascular and renal adverse events were associated, as expected, with nocturnal non-dipping behavior [9, 18, 19]. Predictors of rhythmic components and dipping patterns are different and overlap only in demography and prior comorbidities, further suggesting that these metrics capture different population structures. While a study on a smaller sample of CKD patients (470) showed significantly higher risk for all-cause death, cardiovascular events, and more rapid renal function decline with a high ARV [20], our adjusted model does not show similar results.

A major benefit of quantifying rhythmic components per JTK_CYCLE from BP time series data is its independence from collecting accurate sleep onset and offset data. This non-parametric test has been widely adopted in the circadian clock field to identify oscillating molecular biomarkers [10], thus underscoring the reliability to discover cyclical ordering in timed BP readings. Of the blood pressure determinants listed in Gumz et al. [21], each single one has a diurnal preference tied to mechanisms under circadian control. Signals from the suprachiasmatic nuclei (SCN), entrained to the 24-h environmental cycle, synchronize tissue-specific circadian patterns of clock-controlled gene (CCG) expression through transcriptional-translational loops in the endothelium, vascular smooth muscle cells and the heart. Oscillating Dbp (Albumin D-site-Binding Protein) and Glut4 (glucose transporter 4) gene expression levels drive diurnal fluctuations in vasodilation and heart rate both modulating the diurnal blood pressure phenotype (reviewed in Hastings et al.) [22]. Other sources of diurnal variability related to blood pressure regulation include oscillatory catecholamine levels, vascular adrenergic reactivity, baro-reflex function [23, 24], renin-angiotensin aldosterone system activity [25], cardiac metabolism and response to pro-hypertrophic stimuli [26, 27]. It is a challenge to discern the time-specific contribution of each of these factors to the regulation of the diurnal blood pressure signal. Here, our novel approach of characterizing BP phenotypes for rhythmic components extends the toolbox of established indices (DR, ARV) to parse diurnal BP variability.

A growing body of work suggests that dampening or loss of amplitude in oscillatory processes are associated with aging [28], age-dependent diseases [29, 30], and elevated disease risk [31]. Our findings are in agreement with this framework; however, future efforts are necessary for validation where mechanistic insight drives clinical application. Mouse models with genetically engineered dysfunctional molecular clocks exhibited altered 24-h BP profiles [32]. Vascular stiffening and impaired vascular remodeling have been implicated with a dysregulated matrix metalloproteinase pathway in animal models of clock disruption [33]. This mechanism of accelerated hypertension-mediated end organ damage [34], is a likely factor contributing to the association with death from cardiovascular causes that we detected in the CRIC participants without cyclic blood pressure components.

Wearable cuffless blood pressure devices are being developed and some are currently on the market [35]. These devices aim to provide repeat assessments over longer periods of time, are less disruptive to patients’ sleep and daily activities, and may provide a better reproducible alternative to current cuff-based ABPMs. Refined measures of diurnal blood pressure variation as presented in this study are a powerful tool to analyze the extensive time series data generated by these devices.

Our study has several strengths. We used two well-designed cohorts that characterized participants extensively and followed them up for sufficient periods of time. Both studies also applied standardized procedures for both ABPM measurements and for the adjudication of outcomes. While the sample size is large in the CRIC cohort, the number of participants and events remains smaller in the AASK cohort which limits the power to detect a signal above the noise. Compared to CRIC, the event rate in AASK for cardiovascular death was much lower, suggesting that the cohort disease structure is substantially different. This may be the reason why we were not able to reproduce our CRIC findings among AASK participants. The value, however, lies in synthesizing both approaches to define future clinical study designs. Another reason why our results differ between the two cohorts is that both cohorts used distinct recruitment criteria (Table 1). A crucial difference is the high proportion of diabetics in CRIC (42%) compared to AASK (13%). Circadian autonomic dysfunction, a frequent observation among diabetics [36], could be responsible for an increase in effect size with subsequent detection of a signal above the noise in CRIC but not in AASK. This information could rationalize an initial focus on CKD patients with concomitant cardiovascular and diabetic comorbidities. Also, at the trial phase, the AASK study excluded non-hypertensive kidney disease patients and diabetics. Many of the CRIC cohort patients would not have qualified for participation in the AASK making these two cohorts unique. Furthermore, the high prevalence of CVD among participants who died due to cardiovascular causes (60 out of 83) necessitated a subgroup analysis in patients with prior cardiovascular disease to investigate the association between the loss of rhythmic components and cardiovascular death beyond that which is confounded by prior cardiovascular disease. This decreased the number of cardiovascular deaths in this subgroup analysis leading to a wide confidence interval for our significant association with cardiovascular death. Our dipping category subgroup analysis is also limited by the decreased resulting event numbers; however, it provides necessary results that further differentiate rhythmic components from a calculation of the nocturnal dip. Sleep-disordered breathing such as sleep apnea is known to disrupt sleep-wake rhythms and should also be considered as a source to dampen rhythmic blood pressure components. We were not able to examine this in the present study.

In conclusion, the loss of rhythmic components in blood pressure quantified by the JTK_CYCLE algorithm can be a prognostic indicator of cardiovascular mortality particularly among CKD patients with prior CVD. As wearable blood pressure measurement technologies advance especially in the home environment, such a tool can be valuable in understanding blood pressure rhythms and in identifying patients at higher risk.

## Summary

### What is known about the topic

- Diurnal variability in blood pressure is an independent predictor for cardiovascular risk.
- The non-parametric JTK_CYCLE algorithm is well established to detect rhythmic components in time series datasets.

### What this study adds

- Rhythmic blood pressure components determined by JTK_CYCLE may serve as a potential novel biomarker to unmask excess risk among CKD patients with prior cardiovascular disease.

### Supplementary information

Supplementary Information

## Data Availability

The data from the CRIC Study that support the findings of this study are available upon request at the NIDDK Repository at https://repository.niddk.nih.gov/studies/cric/. The biometric data generated from this study will be made publicly available through the NIDDK Repository and can also be obtained from the CRIC Study with the necessary approvals in place. Instructions for requesting data from the CRIC Study can be found on the CRIC Study website at (http://www.cristudy.org/). The data from the AASK Study that support the findings of this study are available from the NIDDK Repository at https://repository.niddk.nih.gov/studies/aask/.
