# Supplementary material for: Prognostic utility of rhythmic components in 24-h ambulatory blood pressure monitoring for the risk stratification of chronic kidney disease patients with cardiovascular co-morbidity
Source: J Hum Hypertens. 2024 Jan 11;38(5):420–9. doi: 10.1038/s41371-023-00884-0 (PMC11076200; doi:10.1038/s41371-023-00884-0)
Supplement: Supplementary file 1 — Supplementary Information [file 41371_2023_884_MOESM1_ESM.docx]

# **Supplementary Information**

# **Prognostic utility of rhythmic components in 24-hour ambulatory blood pressure monitoring for the risk stratification of chronic kidney disease patients with cardiovascular co-morbidity**

Nadim El Jamal MD^1^, Thomas G. Brooks PhD^1^, Jordana Cohen MD, MSCE^2,3^, Raymond R. Townsend MD^2^, Giselle Rodriguez de Sosa MD^4^, Vallabh Shah PhD, MS^4.5^, Consortium*, Robert G. Nelson MD, PhD^6^, Paul E. Drawz MD, MHS, MS^7^, Panduranga Rao, MBBS^8^, Zeenat Bhat MD^9^, Alexander Chang MD, MS^10^, Wei Yang PhD^3^, Garret A. FitzGerald MD^1,11^ & Carsten Skarke MD^1,11^

^1^ Institute for Translational Medicine and Therapeutics (ITMAT), University of Pennsylvania Perelman School of Medicine, Philadelphia, PA, USA

^2^ Renal-Electrolyte and Hypertension Division, University of Pennsylvania Perelman School of Medicine, Philadelphia, PA, USA

^3^ Department of Biostatistics, Epidemiology and Informatics, University of Pennsylvania Perelman School of Medicine, Philadelphia, PA, USA

^4^ Department of Internal Medicine, School of Medicine, University of New Mexico, Albuquerque, NM, USA

^5^ Department of Biochemistry, School of Medicine, University of New Mexico, Albuquerque, NM, USA

^6^ The Chronic Kidney Disease Section, National Institute of Diabetes and Digestive and Kidney Diseases, National Institutes of Health, Phoenix, AZ, USA

^7^ Division of Nephrology and Hypertension, University of Minnesota, Minneapolis, MN, USA

^8^ Department of Internal Medicine, University of Michigan, Ann Arbor, MI, USA

^9^ Department of Internal Medicine, University of Michigan, Ann Arbor, MI, USA

^10^ Kidney Health Research Institute, Department of Population Health Sciences, Geisinger, Danville, PA, USA

^11^ Department of Medicine, University of Pennsylvania Perelman School of Medicine, Philadelphia, PA, USA

* A list of authors and their affiliations appears at the end of these supplements.

**Short Title:** ABPM Derived Rhythmic Components in CKD

**Corresponding Authors:**

Nadim El Jamal MD ([Nadim.ElJamal@pennmedicine.upenn.edu](mailto:Nadim.ElJamal@pennmedicine.upenn.edu)) & Carsten Skarke, MD ([cskarke@pennmedicine.upenn.edu](mailto:cskarke@pennmedicine.upenn.edu)) Institute for Translational Medicine and Therapeutics (ITMAT), University of Pennsylvania Perelman School of Medicine, Smilow Center for Translational Research 10-101, 3400 Civic Center Blvd, Philadelphia, Pennsylvania 19104, USA

## **Supplementary Figures**

**
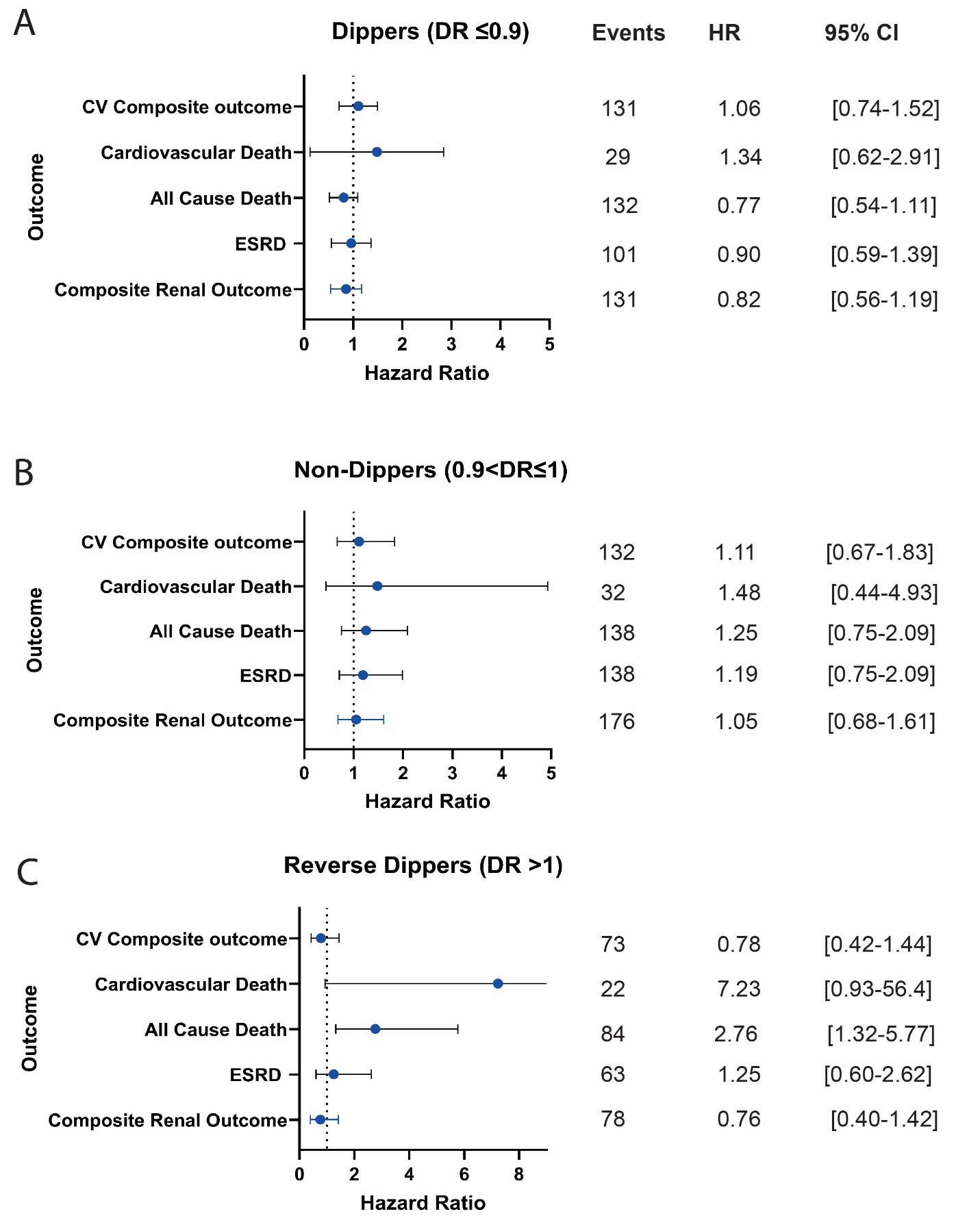
Figure S1:** Hazard ratios for the absence of rhythmic components (JTK p value >0.05) and reaching different outcomes as compared to the retention of rhythmic components (JTK p value ≤0.05) from the CRIC cohort. Adjusted for Age, BMI, Sex, Diabetes, Race, eGFR, Urine Protein to Creatinine Ratio, Clinic SBP, Prior CVD. **A.** Analysis performed only in dippers. **B.** Analysis performed only in non-dippers. **C.** Analysis performed only in reverse dippers. DR: Dipping ratio, CV: Cardiovascular, CVD: Cardiovascular disease, ESRD: End stage renal disease.

## **Supplemental Tables**

**Table S1** Blood pressure profiles of participants in both cohorts. Blood pressure parameters were computed from 24 Hour ABPM (the first 24 Hour ABPM in AASK).

|  | **CRIC**  **N(%)** | **AASK**  **N(%)** |
| --- | --- | --- |
| **Rhythmic Components present (JTK p ≤0.05)** | 514 (34%) | 169 (26%) |
| **Rhythmic Components absent (JTK p >0.05)** | 988 (66%) | 474 (74%) |
| **Dipper (DR ≤0.9)** | 678 (45%) | 127 (20%) |
| **Dipper w/ rhythmic components (DR ≤0.9& JTK<0.05)** | 376 (25%) | 54 (8%) |
| **Non-Dipper (0.9<DR≤1)** | 572 (38%) | 266 (41%) |
| **Non-Dipper w/ rhythmic components (0.9<DR≤1& JTK<0.05)** | 92 (6%) | 38 (6%) |
| **Reverse Dipper (DR>1)** | 252 (17%) | 250 (39%) |
| **Reverse Dipper w/ rhythmic components (DR>1 & JTK<0.05)** | 46 (3%) | 77 (12%) |
| **Controlled BP (24 Hr Mean<125/75)** | 549 (37%) | 100 (16%) |
| **Uncontrolled BP (24 Hr Mean ≥125/75)** | 953 (63%) | 543 (85%) |

**Table S2.** Multivariate logistic regression for non-dipping status (dipping ratio >0.9) among CRIC cohort participants. All variables in the table were included as covariates in the model. eGFR: estimated glomerular filtration rate by the CRIC cohort equation. BP: blood pressure. PCR: urine protein to creatinine ratio.

| **Covariate** | **Odds Ratio** | **95%CI Lower** | **95%CI Upper** | | **P-value** | |
| --- | --- | --- | --- | --- | --- | --- |
| Age <45 | Reference |  | |  | |  |
| Age From 45 to <65 | 1.32 | 0.8 | | 2.18 | | 0.276 |
| Age 65+ | 1.17 | 1.01 | | 2.78 | | 0.047 |
| BMI <25 | Reference |  | |  | |  |
| BMI 25 to <30 | 1.07 | 0.74 | | 1.55 | | 0.705 |
| BMI ≥30 | 1.27 | 0.89 | | 1.8 | | 0.182 |
| eGFR >60 ml/min/1.73m^2^ | Reference |  | |  | |  |
| eGFR: 30 to <60 ml/min/1.73m^2^ | 1.11 | 0.82 | | 1.51 | | 0.483 |
| eGFR<30 ml/min/1.73m^2^ | 1.46 | 0.99 | | 2.15 | | 0.059 |
| Proteinuria (PCR <150mg/g) | Reference |  | |  | |  |
| Proteinuria (PCR: 150-500 mg/g) | 1.08 | 0.8 | | 1.46 | | 0.623 |
| Proteinuria (PCR>500 mg/g) | 1.56 | 1.12 | | 2.17 | | 0.009 |
| Controlled BP | Reference |  | |  | |  |
| Un-Controlled BP (Mean 24 Hr BP ≥ 125/75mmHg) | 1.64 | 1.27 | | 2.12 | | <0.001 |
| Male Sex | Reference |  | |  | |  |
| Female Sex | 0.85 | 0.67 | | 1.09 | | 0.195 |
| Non-Diabetic | Reference |  | |  | |  |
| Diabetic | 1.23 | 0.96 | | 1.67 | | 0.1 |
| Race: White | Reference |  | |  | |  |
| Race: Black | 1.55 | 1.2 | | 2.01 | | 0.001 |
| Race: Other | 0.92 | 0.63 | | 1.32 | | 0.64 |
| Prior CVD: No | Reference |  | |  | |  |
| Prior CVD: Yes | 1.58 | 1.23 | | 2.03 | | <0.001 |

**Table S3.** Mixed effects logistic regression for non-dipping status (dipping ratio >0.9 among AASK cohort participants. All variables in the table were included as covariates in the model. eGFR: estimated glomerular filtration rate by the 2021 CKD-EPI equation. PCR: urine protein to creatinine ratio. BP: blood pressure. ACE I: angiotensin converting enzyme inhibitor. CCB: calcium channel blocker.

| **Covariate** | **Odds Ratio** | **95%CI Lower** | **95%CI Upper** | **P-value** |
| --- | --- | --- | --- | --- |
| Age <45 | Reference |  |  |  |
| Age From 45 to <65 | 1.4 | 0.5 | 0.39 | 0.52 |
| Age 65+ | 1.62 | 0.57 | 4.6 | 0.368 |
| BMI <25 | Reference |  |  |  |
| BMI 25 to <30 | 0.78 | 0.41 | 1.48 | 0.446 |
| BMI ≥30 | 1.25 | 0.66 | 2.38 | 0.491 |
| eGFR >60 ml/min/1.73m^2^ | Reference |  |  |  |
| eGFR: 30 to <60 ml/min/1.73m^2^ | 1.94 | 0.68 | 5.59 | 0.218 |
| eGFR<30 ml/min/1.73m^2^ | 1.88 | 0.63 | 5.63 | 0.256 |
| Proteinuria (PCR <150mg/g) | Reference |  |  |  |
| Proteinuria (PCR: 150-500 mg/g) | 1.16 | 0.64 | 2.09 | 0.626 |
| Proteinuria (PCR>500 mg/g) | 1.08 | 0.6 | 1.93 | 0.803 |
| Controlled BP | Reference |  |  |  |
| Un-Controlled BP (Mean 24 Hr BP ≥ 125/75mmHg) | 2.02 | 1.23 | 3.31 | 0.006 |
| Male Sex | Reference |  |  |  |
| Female Sex | 0.7 | 0.44 | 1.12 | 0.14 |
| Non-Diabetic | Reference |  |  |  |
| Diabetic | 0.53 | 0.28 | 0.99 | 0.048 |
| Prior CVD: No | Reference |  |  |  |
| Prior CVD: Yes | 1.19 | 0.74 | 1.91 | 0.467 |
| Drug Randomization Group: ACE I | Reference |  |  |  |
| Drug Randomization Group: Beta Blocker | 1.86 | 1.12 | 3.1 | 0.016 |
| Drug Randomization Group: CCB | 0.96 | 0.51 | 1.83 | 0.91 |
| BP Target Randomization Group: Lower Target (MAP <92) | Reference |  |  |  |
| BP Target Randomization Group: Usual Target (MAP 102-107) | 1.29 | 0.82 | 2.03 | 0.269 |
| Time from initial ABPM (years) | 0.996 | 0.89 | 1.12 | 0.96 |

**Table S4.**  Cross Tabulation of dipping status and the presence or absence of rhythmic components in ABPM among participants with prior cardiovascular disease who died due to cardiovascular causes.

|  | **Rhythmic Components Present** | **Rhythmic Components Absent** | **Total** |
| --- | --- | --- | --- |
| **Dipper** | 6 | 14 | 20 |
| **Non-Dipper** | 0 | 24 | 24 |
| **Reverse-Dipper** | 0 | 21 | 21 |
| ***Total*** | *6* | *59* | *65* |

**Table S5.** Hazard ratios for the different dipping categories and reaching different outcomes from the CRIC cohort. Adjusted for Age, BMI, Sex, Diabetes, Race, eGFR, Urine Protein to Creatinine Ratio, Clinic SBP, Prior CVD. DR: Dipping ratio, CV: Cardiovascular, CVD: Cardiovascular disease, ESRD: End stage renal disease.

|  |  | **Unadjusted Model** | | | **Adjusted Model** | | |
| --- | --- | --- | --- | --- | --- | --- | --- |
| **Outcome** | **Dipping Category** | **Hazard Ratio** | **95% CI** | **p-value** | **Hazard Ratio** | **95% CI** | **p-value** |
| **Composite Renal Outcome** | Dipper | Reference |  |  |  |  |  |
|  | Non-Dipper | 1.81 | 1.47-2.23 | <0.001 | 1.27 | 1.01-1.61 | 0.043 |
|  | Reverse Dipper | 2.99 | 1.53-2.60 | <0.001 | 1.39 | 1.03-1.87 | 0.032 |
| **ESRD** | Dipper | Reference |  |  |  |  |  |
|  | Non-Dipper | 1.8 | 1.42-2.28 | <0.001 | 1.07 | 0.82-1.41 | 0.614 |
|  | Reverse Dipper | 1.97 | 1.47-2.66 | <0.001 | 1.37 | 0.98-1.91 | 0.064 |
| **All Cause Death** | Dipper | Reference |  |  |  |  |  |
|  | Non-Dipper | 1.4 | 1.12-1.74 | 0.003 | 0.93 | 0.73-1.19 | 0.557 |
|  | Reverse Dipper | 2.44 | 1.91-3.12 | <0.001 | 1.14 | 0.85-1.53 | 0.368 |
| **Cardiovascular Death** | Dipper | Reference |  |  |  |  |  |
|  | Non-Dipper | 1.3 | 0.80-2.12 | 0.297 | 0.99 | 0.60-1.65 | 0.998 |
|  | Reverse Dipper | 2.55 | 1.52-4.28 | <0.001 | 1.13 | 0.62-2.06 | 0.694 |
| **CV Composite outcome** | Dipper | Reference |  |  |  |  |  |
|  | Non-Dipper | 1.35 | 1.07-1.69 | 0.01 | 0.93 | 0.73-1.19 | 0.564 |
|  | Reverse Dipper | 2.09 | 1.61-2.72 | <0.001 | 1.01 | 0.75-1.37 | 0.929 |

**Table S6.** Hazard ratios for the different dipping categories and reaching different outcomes from the AASK cohort. Adjusted for Age, BMI, Sex, Diabetes, eGFR, Urine Protein to Creatinine Ratio, Clinic SBP, , Prior CVD Drug and blood pressure target groups randomized to in the prior trial. DR: Dipping ratio, CV: Cardiovascular, CVD: Cardiovascular disease, ESRD: End stage renal disease.

|  |  | **Unadjusted Model** | | | **Adjusted Model** | | |
| --- | --- | --- | --- | --- | --- | --- | --- |
| **Outcome** | **Dipping Category** | **Hazard Ratio** | **95% CI** | **p-value** | **Hazard Ratio** | **95% CI** | **p-value** |
| **Composite Renal Outcome** | Dipper | Reference |  |  |  |  |  |
|  | Non-Dipper | 1.05 | 0.69-1.61 | 0.816 | 0.79 | 0.43-1.46 | 0.452 |
|  | Reverse Dipper | 1.10 | 0.72-1.70 | 0.652 | 1.25 | 0.69-2.25 | 0.465 |
| **ESRD** | Dipper | Reference |  |  |  |  |  |
|  | Non-Dipper | 0.94 | 0.57-1.58 | 0.827 | 0.78 | 0.37-1.63 | 0.505 |
|  | Reverse Dipper | 0.98 | 0.58-1.64 | 0.930 | 1.38 | 0.66-2.88 | 0.387 |
| **All Cause Death** | Dipper | Reference |  |  |  |  |  |
|  | Non-Dipper | 0.94 | 0.52-1.72 | 0.851 | 0.82 | 0.40-1.72 | 0.606 |
|  | Reverse Dipper | 1.70 | 0.97-2.98 | 0.065 | 1.31 | 0.67-2.56 | 0.437 |
| **Cardiovascular Death** | Dipper | Reference |  |  |  |  |  |
|  | Non-Dipper | 1.06 | 0.32-3.45 | 0.956 | 1.7 | 0.45-6.53 | 0.436 |
|  | Reverse Dipper | 2.39 | 0.81-7.06 | 0.133 | 2.08 | 0.57-7.57 | 0.268 |
| **CV Composite outcome:** | Dipper | Reference |  |  |  |  |  |
|  | Non-Dipper | 1.41 | 0.80-2.49 | 0.236 | 1.58 | 0.51-3.07 | 0.179 |
|  | Reverse Dipper | 1.79 | 1.02-3.14 | 0.043 | 1.59 | 0.82-3.08 | 0.171 |

**Table S7.** Hazard ratios for the different average real variability (ARV) tertials and reaching different outcomes from the CRIC cohort. Adjusted for Age, BMI, Sex, Diabetes, Race, eGFR, Urine Protein to Creatinine Ratio, Clinic SBP, , Prior CVD. CV: Cardiovascular, CVD: Cardiovascular disease, ESRD: End stage renal disease.

|  |  | **Unadjusted Model** | | | **Adjusted Model** | | |
| --- | --- | --- | --- | --- | --- | --- | --- |
| **Outcome** | **ARV Category** | **Hazard Ratio** | **95% CI** | **p-value** | **Hazard Ratio** | **95% CI** | **p-value** |
| **Composite Renal Outcome** | ≤9 | Reference |  |  |  |  |  |
|  | >9, ≤11 | 1.18 | 0.93-1.49 | 0.165 | 1.02 | 0.79-1.32 | 0.863 |
|  | >11 | 1.47 | 1.17-1.85 | 0.001 | 0.95 | 0.73-1.25 | 0.728 |
| **ESRD** | ≤9 | Reference |  |  |  |  |  |
|  | >9, ≤11 | 1.11 | 0.85-1.45 | 0.44 | 1.04 | 0.77-1.39 | 0.82 |
|  | >11 | 1.49 | 1.16-1.93 | 0.002 | 0.97 | 0.71-1.34 | 0.861 |
| **All Cause Death** | ≤9 | Reference |  |  |  |  |  |
|  | >9, ≤11 | 1.43 | 1.12-1.84 | 0.004 | 1.12 | 0.85-1.47 | 0.437 |
|  | >11 | 1.8 | 1.42-2.29 | <0.001 | 1.14 | 0.87-1.50 | 0.335 |
| **Cardiovascular Death** | ≤9 | Reference |  |  |  |  |  |
|  | >9, ≤11 | 1.23 | 0.72-2.10 | 0.451 | 0.91 | 0.52-1.61 | 0.749 |
|  | >11 | 1.64 | 0.99-2.72 | 0.057 | 0.92 | 0.53-1.58 | 0.76 |
| **CV Composite outcome:** | ≤9 | Reference |  |  |  |  |  |
|  | >9, ≤11 | 1.45 | 1.12-1.89 | 0.006 | 1.23 | 0.92-1.63 | 0.158 |
|  | >11 | 2.05 | 1.60-2.64 | <0.001 | 1.29 | 0.98-1.70 | 0.074 |

## **CRIC Consortium**

- Amanda H. Anderson PhD, MPH
  - multi-PI for the Scientific and Data Coordinating Center
  - University of Alabama at Birmingham
- Lawrence J. Appel, MD, MPH
  - Johns Hopkins University ProHealth
  - CRIC Principal Investigator
- Jing Chen, MD, MMSc, MSc
  - Tulane University School of Medicine
  - CRIC Principal Investigator
- Debbie L Cohen, MD
  - University of Pennsylvania
  - CRIC Principal Investigator
- Laura M Dember, MD
  - University of Pennsylvania
  - PI for the Scientific and Data Coordinating Center
- Alan S. Go, MD
  - Kaiser Permanente of Northern California
  - CRIC Principal Investigator
- James P. Lash, MD
  - University of Illinois at Chicago
  - CRIC Principal Investigator
- Robert G. Nelson, MD, PhD, MS
  - National Institute of Diabetes and Digestive and Kidney Diseases, National Institutes of Health (NIDDK-NIH)
  - CRIC Principal Investigator
- Mahboob Rahman, MD
  - University Hospitals Cleveland Medical Center
  - CRIC Principal Investigator
- Panduranga S. Rao, MD
  - University of Michigan
  - CRIC Principal Investigator
- Vallabh O. Shah, PhD, MS
  - University of New Mexico
  - CRIC Principal Investigator
- Mark L. Unruh, MD, MS
  - University of New Mexico
  - CRIC Principal Investigator
